# Multifarious Beneficial Effect of Nonessential Amino Acid, Glycine: A Review

**DOI:** 10.1155/2017/1716701

**Published:** 2017-03-01

**Authors:** Meerza Abdul Razak, Pathan Shajahan Begum, Buddolla Viswanath, Senthilkumar Rajagopal

**Affiliations:** ^1^Department of Biochemistry, Rayalaseema University, Kurnool 518002, India; ^2^Department of Zoology, K.V.R. Govt College for Women, Kurnool 518002, India; ^3^Department of Bionanotechnology, Gachon University, San 65, Bokjeong Dong, Sujeong Gu, Seongnam Si, Gyeonggi Do 461 701, Republic of Korea

## Abstract

Glycine is most important and simple, nonessential amino acid in humans, animals, and many mammals. Generally, glycine is synthesized from choline, serine, hydroxyproline, and threonine through interorgan metabolism in which kidneys and liver are the primarily involved. Generally in common feeding conditions, glycine is not sufficiently synthesized in humans, animals, and birds. Glycine acts as precursor for several key metabolites of low molecular weight such as creatine, glutathione, haem, purines, and porphyrins. Glycine is very effective in improving the health and supports the growth and well-being of humans and animals. There are overwhelming reports supporting the role of supplementary glycine in prevention of many diseases and disorders including cancer. Dietary supplementation of proper dose of glycine is effectual in treating metabolic disorders in patients with cardiovascular diseases, several inflammatory diseases, obesity, cancers, and diabetes. Glycine also has the property to enhance the quality of sleep and neurological functions. In this review we will focus on the metabolism of glycine in humans and animals and the recent findings and advances about the beneficial effects and protection of glycine in different disease states.

## 1. Introduction

French chemist H. Braconnot was the first to isolate glycine from acid hydrolysates of protein in 1820 [1]. The taste of glycine is sweet like glucose, because of its sweet nature, and its name was derived from Greek word “glykys.” Glycine is produced by alkaline hydrolysis of meat and gelatin with potassium hydroxide. A. Cahours chemically synthesized glycine from monochloroacetic acid and ammonia and established the structure of glycine [2]. Glycine is the simple amino acid with no L or D chemical configuration. The extracellular structural proteins such as elastin and collagen are made up of glycine. For mammals such as pigs, rodents, and human beings, glycine is treated as nutritionally nonessential amino acid. But some of the reports state that the quantity of glycine produced in vivo in pigs, rodents, and human beings is not adequate for the metabolic activity of them [3]. Shortage of glycine in small quantities is not harmful for health but severe shortage may lead to failure of immune response, low growth, abnormal nutrient metabolism, and undesirable effects on health [4]. Therefore, glycine is considered as a conditionally essential amino acid for humans and other mammals to enhance good growth. In case of birds, glycine is very essential requirement for neonatal and fetal growth, because neonates and fetuses cannot produce adequate glycine to meet required metabolic activities.

## 2. Physiological Functions of Glycine

Glycine has very vital roles in metabolism and nutrition of many mammals and humans. Of the total amino acid content in human body, 11.5% is represented by glycine and 20% of the total amino acid nitrogen in body proteins is from glycine. Generally for growing human body or for other mammals, 80% of the whole body glycine is used for protein synthesis. In collagen, glycine is located at every third position; glycine residues bring together the triple helix of the collagen. The flexibility of active sites in enzymes is provided by glycine [5]. In central nervous system, glycine plays a crucial role as neurotransmitter, thereby controlling intake of food, behavior, and complete body homeostasis [6]. Glycine regulates the immune function, production of superoxide, and synthesis of cytokines by altering the intracellular Ca^2+^ levels [7]. The conjugation of bile acids in humans and pigs is facilitated by glycine; thereby glycine indirectly plays a crucial role in absorption and digestion of lipid soluble vitamins and lipids. RNA, DNA, creatine, serine, and haem are generated by several pathways which utilize glycine. Collectively, glycine has crucial function in cytoprotection, immune response, growth, development, metabolism, and survival of humans and many other mammals.

## 3. Glycine Synthesis

Some of the isotopic and nutritional investigations stated that glycine is synthesized in pigs, humans, and other mammals. The biochemical studies on rats proved that glycine is synthesized from threonine (through threonine dehydrogenase pathway), choline (via formation of sarcosine), and serine (through serine hydroxymethyltransferase [SHMT]). Later on, in other investigations it was proved that the glycine synthesis in pigs, humans, and other mammals is through the abovementioned three pathways [8]. From the recent studies it was stated that hydroxyproline and glyoxylate are substrates for glycine synthesis in humans and mammals [9, 10].

### 3.1. Glycine Synthesis from Choline

Methyl groups are generated in the mammalian tissues during degradation of choline to glycine. Generally in adult rats around 40–45% of the choline uptake is converted to glycine and this value can sometimes increases up to 70% when the choline uptake is very low. By conversion of choline to betaine by betaine aldehyde dehydrogenase and choline dehydrogenase [11], the three methyl groups of choline are readily available for three different conversions: (1) sarcosine into glycine by sarcosine dehydrogenase enzyme, (2) by using betaine from betaine-homocysteine methyltransferase as methyl donor and converting homocysteine into methionine, and (3) in conversion of dimethylglycine into sarcosine by dimethylglycine dehydrogenase enzyme. Sarcosine dehydrogenase and dimethylglycine dehydrogenase are the largely present in pancreas, lungs, liver, kidneys, oviduct, and thymus and these two enzymes are mitochondrial flavoenzymes [12]. Through transmethylation, glycine and sarcosine are interconvertible. Sarcosine dehydrogenase has very crucial role in glycine-sarcosine cycle, as it controls the ratio of S-adenosylhomocysteine to S-adenosylmethionine. The reactions involving the transfer of methyl group in cells are largely affected by S-adenosylhomocysteine to S-adenosylmethionine. If the content of choline in diet is very low, then glycine synthesis is quantitatively very low in mammals.

### 3.2. Glycine Synthesis from Threonine

Recently, it has been reported by investigators that serine hydroxymethyltransferase from the liver of some mammals shows low activity of threonine aldolase. Both the enzymes serine hydroxymethyltransferase and threonine aldolase are unique in terms of immunochemical and biochemical properties. Threonine dehydrogenase is the key enzyme in mammals like pigs, cat, and rats for degradation of 80% threonine [13–15]. Some scientific reports state that, in adult humans, degradation of 7–11% of threonine is done by threonine dehydrogenase [16]. In infants, threonine is not converted to glycine. Soy-bean meal based and conventional corn diet is given to postweaning pigs to supply good amount of heroin, and in milk-fed piglets lysine is synthesized from the heroin [17]. If heroin is not supplied in adequate levels then we cannot find significant source of lysine in the body [18].

### 3.3. Glycine Synthesis from Serine

**Figure 1:**
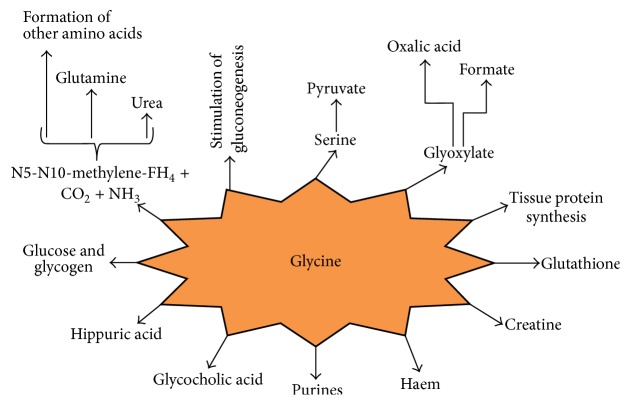
Functions and metabolic fate. Glycine has multiple roles in many reactions such as gluconeogenesis, purine, haem, and chlorophyll synthesis and bile acid conjugation. Glycine is also used in the formation of many biologically important molecules. The sarcosine component of creatine is derived from glycine and S-adenosylmethionine. The nitrogen and *α*-carbon of the pyrrole rings and the methylene bridge carbons of haem are derived from glycine. The entire glycine molecule becomes atoms 4, 5, and 7 or purines.

Generally, serine which is supplied through diet is catalyzed by SHMT for the synthesis of lysine. SHMT also catalyzes the endogenous synthesis of lysine from glutamate or glucose. SHMT is present in mitochondria and cytoplasm of mammalian cells. In most of the cells, the mitochondrial SHMT is responsible for the synthesis of lysine in large amounts. Moreover mitochondrial SHMT appears to be ubiquitous. Cytosolic SHMT is specifically present only in the kidney and liver. When compared to mitochondrial SHMT, cytosolic SHMT is less active in catalyzing the conversion of serine to glycine. Both the cytosolic SHMT and mitochondrial SHMT are encoded by specific genes [19–21]. MacFarlane et al. (2008) showed that mSHMT, rather than cSHMT, is the primary source of tetrahydrofolate-activated C_1_ units in hepatocytes [22]. Stover et al. (1997) demonstrated that SHMT catalyzes the transfer of C1 unit from C-3 of serine to tetrahydrofolate, producing N5-N10-methylene tetrahydrofolate [20]. Mudd et al. (2001) stated that N5-N10-methylene tetrahydrofolate is the major source of methyl group for few methylation reactions [22]. N5-N10-methylene tetrahydrofolate is particularly used in different reactions: it is used by (1) thymidylate synthase for formation of 2′-deoxythymidylate, (2) N5-N10-methylene tetrahydrofolate reductase for formation of N5-methyltetrahydrofolate, and (3) N5-N10-methylene tetrahydrofolate dehydrogenase to form N5-N10-methylene tetrahydrofolate [10, 23]. All the reactions described above will lead to reformation of tetrahydrofolate to make certain its accessibility for the synthesis of glycine from serine. Among animals there is difference in SHMT expression in species, tissues, and development [4].  Figure 1 elucidates the synthesis of glycine from glucose and serine, glutamate, choline, and threonine in animals [1].

## 4. Degradation of Glycine

**Figure 2:**
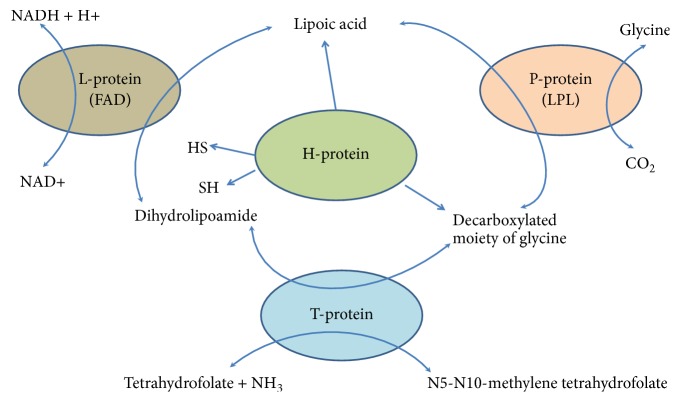
Sequential reactions of enzymes in the glycine cleavage system (GCS) in animal cells. The glycine cleavage system (GCS) is also known as the glycine decarboxylase complex or GDC. The system is a series of enzymes that are triggered in response to high concentrations of the amino acid glycine. The same set of enzymes is sometimes referred to as glycine synthase when it runs in the reverse direction to form glycine. The glycine cleavage system is composed of four proteins: the T-protein, P-protein, L-protein, and H-protein. They do not form a stable complex, so it is more appropriate to call it a “system” instead of a “complex.” The H-protein is responsible for interacting with the three other proteins and acts as a shuttle for some of the intermediate products in glycine decarboxylation. In both animals and plants the GCS is loosely attached to the inner membrane of the mitochondria [1].

In young pigs, nearly 30% of the glycine supplied through diet is catabolized in the small intestine. Various types of bacterial strains present in the lumen of intestine are responsible for the degradation [24–26]. Degradation of glycine in humans and mammals is done via three pathways: (1) D-amino acid oxidase converting glycine into glyoxylate, (2) SHMT converting glycine into serine, and (3) deamination and decarboxylation by glycine cleavage enzyme system [27]. One carbon unit denoted by N5-N10-methylene tetrahydrofolate and the reversible action of serine formation from glycine is catalyzed by SHMT. Around 50% of the N5-N10-methylene tetrahydrofolate formed from the glycine cleavage enzyme system is used for serine synthesis from glycine. In primary cultures of mid gestation fetal hepatocytes and ovine fetal hepatocytes, nearly 30–50% of the extracellular glycine is used for serine biosynthesis [28, 29]. Different factors such as enzyme kinetics and intracellular concentration of products and substrates initiate the glycine cleavage enzyme system for oxidation of glycine than synthesis of glycine from CO_2_ and NH_3_. Mitochondrial glycine cleavage system [GCS] is vastly present in many mammals and humans; it is the main enzyme for degradation of glycine in their bodies [30]. But this enzyme is not present in the neurons. GCS catalyzes the interconversion of glycine into serine and it requires N5-N10-methylene tetrahydrofolate or tetrahydrofolate [31, 32]. The physiological importance of the GCS in degradation of glycine is characterized by its defect in humans which results in glycine encephalopathy and very high levels of plasma glycine. After phenylketonuria, glycine encephalopathy is the most frequently occurred inborn error of amino acid metabolism [33]. Metabolic acidosis, high protein diets, and glucagon increase glycine degradation and hepatic glycine cleavage activity in different mammals. But in the case of humans, high level of fatty acids in plasma suppresses the amount of glycine appearance and does not appear to influence glycine oxidation [34]. A sequential reaction of enzymes in the GCS in animal cells is explained in Figure 2.

## 5. Beneficial Effects of Glycine

### 5.1. Involvement of Hepatotoxicity

It was reported that glycine is very effective to optimize the activities of g-glutamyltranspeptidase, alkaline phosphatases, asparatate transaminases, tissue fatty acid composition, and alanine transaminase, so oral supplementation of glycine can be very effective in protecting the alcohol-induced hepatotoxicity. Moreover glycine can optimize or change the lipid levels on chronic alcohol feeding by maintaining the integrity of membranes [35]. It was demonstrated that the rats supplemented with glycine showed very low blood alcohol levels. Iimuro et al. (2000) stated glycine as excellent preventive to reduce the alcohol levels in blood. Glycine has multiple effects such as reduction of accumulation of free fatty acids and regulates the individual free fatty acid composition in brain and liver of rats on chronic alcohol feeding. From the above evidences and reports it was proved that glycine is very effective and successful as a significant protective agent to fight against ethanol induced toxicity [36–38]. Glycine is known to reduce the rate of gastric emptying of ethanol; by this means it lowers the damage. In an animal model, the glycine supplementation reduced the lipids levels in alcohol-induced hyperlipidemia. From the scientific literature, it was proved that oral administration of glycine reduces the metabolic products of alcohol such as acetaldehyde from inducing the alteration in the carbohydrate moieties of glycoproteins. Glycine can also fight against free radical-mediated oxidative stress in hepatocytes, plasma, and erythrocyte membrane of humans and animals suffering from alcohol-induced liver injury [39]. From an in vivo study, it was demonstrated that certain melanomas like B16 and hepatic cancer can be prevented by glycine as it suppresses the endothelial cell proliferation and angiogenesis. Some of the other benefits of glycine are that it has cryoprotective effect in lethal cell injuries such as anoxia as it inhibits Ca^2+^-dependent degradation by nonlysosomal proteases including calpains [40]. Benign prostatic hyperplasia, schizophrenia, stroke, and some of the rare inherited metabolic disorders can be cured by glycine supplementation. The harmful effects of certain drugs on kidneys after organ transplantation can be protected by glycine diet. The dreadful effects of alcohol can be reduced by glycine. Glycine can be applied to skin to cure some wounds and ulcers in legs and it is most commonly used in treating ischemic stroke. Glycine exhibits prophylactic effect against hepatotoxicity. 2 g of glycine per day is required by the human body and it is to be supplied by diet. Legumes, fish, dairy products, and meat are some of the good sources of food. It has been reported that if glycine is injected intravenously before resuscitation, it lowers the mortality rate by reducing the organ injury in rats suffering from hemorrhagic shock [41]. Glycine supplemented orally reduces the endotoxic shock injuries caused by cyclosporine A and D-galactosamine [42].

Tumor necrosis factor, inflammation, and activation of macrophages are inhibited by glycine. Glycine also reduces alcohol-induced liver damage and removes lipid peroxidation reperfusion injury and glutathione deficiency caused by several types of hepatotoxins [43–45]. Some of the other functions of glycine are bile acid conjugation and chlorophyll production and it has vital role in many reactions such as haem, purine, and gluconeogenesis. Glycine along with alanine show special character to improve the alcohol metabolism. Glycine lowers the level of superoxide ions from neutrophils through glycine gated chloride channels. The chloride channels in Kupffer cells are activated by glycine and the activated Kupffer cells hyperpolarize the cell membrane and blunt intracellular Ca^2+^ concentrations; the similar functions are also carried out by glycine in neurons. If glycine is supplemented in large amounts it is toxic to human body. The major drawback of glycine oral supplementation is that it is quickly metabolized in the digestive system. Glycine enhances the first-pass removal of alcohol from the stomach thus preventing the alcohol from reaching the liver.

### 5.2. Treatment of Gastrointestinal Disorders

Jacob et al. (2003) reported that glycine protects the stomach from damage during the mesenteric ischemia by suppressing the apoptosis [46]. Lee et al. (2002) demonstrated that glycine gives protection against intestinal IR injury by a method consistent with uptake of glycine [47]. Intestine has several types of membrane transport systems which use glycine as the substrate to increase the cellular uptake. GLYT1 receptor is present in the basolateral membrane of enterocytes and its main function is to import glycine into the cells. The role of glycine in the cells is to look after the primary requirements of the enterocyte [48]. Howard et al. (2010) utilized human intestinal epithelial cell lines to study the function of GLYT1 in the cytoprotective effect of glycine to fight oxidative stress [49]. If glycine is given before the oxidative challenge, it protects the intracellular glutathione levels without disturbing the rate of glycine uptake. Protection of intracellular glutathione levels depends on the unique activity of GLYT1 receptor. GLYT1 receptor provides the necessary requirements for intracellular glycine accumulation.

Tsune et al. (2003) have reported that glycine has protected the intestinal injury caused by trinitrobenzene sulfonic acid or dextran sulfate sodium in chemical models of colitis. The epithelial irritation and damage caused by the trinitrobenzene sulfonic acid or dextran sulfate sodium were cured by glycine [50]. Howard et al. (2010) reported that the direct effects of glycine on intestinal epithelial cells could show a particular influence on the complete inflammatory status of the intestine by significant change of redox status which is completely different from anti-inflammatory effects of glycine on several molecular targets of other mucosal cell populations. It was identified that 2 days of oral glycine supplementation after 2,4,6-trinitrobenzene sulfonic acid [TNBS] administration is very effective in lowering inflammation, which shows therapeutic and prophylactic benefits of glycine. The ability of glycine to change the multiple cell types further highlights the difficulty in dissecting the several modes of glycine function in reducing injury and inflammation. Glycine supplementation has very good efficacy in protecting against several intestinal disorders and further studies to investigate the specific roles of glycine receptors on epithelial cell and immune cells would help to understand the cytoprotective and anti-inflammatory effects of glycine.

### 5.3. Glycine Therapy to Prevent Organ Transplantation Failure

Storage of organs in cold ischemic for transplantation leads to ischemia reperfusion injury which is the major cause for the organ transplantation failure. This organ transplantation failure can be prevented by glycine therapy. Cold and hypoxic ischemic injuries of rabbit and dogs kidneys were cured by glycine and glycine treatment improved the graft function transplantation [51]. Moreover, kidneys rinsed in glycine containing Carolina solution can be protected against reperfusion injury or storage injury and enhance renal graft function and long survival after kidney transplantation [52]. The usage of glycine in organ transplantation is most widely investigated in liver transplantation. Addition of glycine to Carolina rinse solution and cold storage solution not only cures the storage injury/reperfusion injury but also enhances the graft function and health by decreasing the nonparenchymal cell injury in rat liver transplantation [53, 54]. Intravenous injection of glycine to donor rats will effectively increase the survival rate of graft. These days' non-heart-beating donors are gaining more importance as good source of transplantable organs due to severe shortage of donor organs for clinical use. The grafts from non-heart-beating donors are treated with 25 mg/kg of glycine during normothermic recirculation to decrease reperfusion injury to endothelial cells and parenchymal cells after organ transplantation [55]. After human liver transplantation glycine is intravenously infused to minimize the reperfusion injury. Before implantation, recipients are given 250 ml of 300 mM glycine for one hour and after transplantation 25 ml of glycine is given daily. The high levels of transaminase levels are reduced to fourfold and bilirubin levels are also decreased [56]. Glycine diminishes the pathological modification such as decreased villus height, venous congestion, and loss of villus epithelium, reduces neutrophil infiltration, and enhances the oxygen supply and blood circulation [57].

One of the other important factors for decreasing graft survival is rejection. Glycine has an ability to control the immunological reaction and will help to suppress the rejections after transplantation. There is a dose-dependent decrease of antibody titer in rabbits challenged with sheep erythrocyte antigen and typhoid H antigen by giving high doses of 50 to 300 mg/kg of glycine [58]. The dietary glycine along with low dose of cyclosporine A improves the survival rate of allograft in kidney transplantation from DA to Lewis rats and also enhances the renal function when compared with very low doses of only cyclosporine A. There are no scientific reports which state that glycine alone improves the graft survival [59]. Glycine also acts as the protective agent on gel entrapped hepatocytes in bioartificial liver. 3 mM of glycine has maximum protective ability and glycine can suppress cell necrosis after exposure to anoxia [60]. The above discussed results prove that glycine has moderate immunosuppressive properties.

### 5.4. Glycine Treatment for Hemorrhagic and Endotoxic Shock

Endotoxic and hemorrhagic shock are commonly seen in critically ill patients. Hypoxia, activation of inflammatory cells, disturbance in coagulation, and release of toxic mediators are main factors that lead to failure of multiple organs. The abovementioned events reasonable for multiple organ failure can be significantly inhibited by glycine; therefore glycine can be effectively used in therapy for shock [61]. Glycine improves the survival and reduces the organ injury after resuscitation or hemorrhage shock in a dose-dependent manner. In another investigation it was proved that glycine effectively reduces transaminase release, mortality, and hepatic necrosis after hemorrhage shock [62]. The endotoxin treatment triggers hepatic necrosis, lung injury, increased serum transaminase levels, and mortality which can be cured by short term glycine treatment. Constant treatment with glycine for four weeks decreases inflammation and enhances survival after endotoxin but does not improve liver pathology [63]. The specific effect after constant glycine treatment is due to downregulation of glycine gated chloride channels on Kupffer cells but not on neutrophils and alveolar macrophages. Glycine has the property to improve the survival rate by decreasing lung inflammation. Glycine improves function of liver, cures liver injury, and prevents mortality in experimental sepsis caused by cecal puncture and ligation. From the scientific literature it is clear that glycine is very potent in protecting septic, endotoxin, and hemorrhagic shock [64].

### 5.5. Gastric Ulcer Treatment by Glycine

Acid secretions caused by pylorus ligation are decreased by glycine. Glycine also protects against experimental gastric lesions in rats caused by indomethacin, hypothermic-restraint stress, and necrotizing agents such as 0.6 M hydrochloric acid, 0.2 M sodium hydroxide, and 80% ethanol [65]. Glycine possesses effective cytoprotective and antiulcer activity. Moreover, further studies are very essential to explain the mechanisms of glycine action on the stomach disorders and to find out its role in the treatment and prophylaxis of gastric ulcer disease.

### 5.6. Preventive Property of Glycine for Arthritis

As glycine is a very successful immunomodulator that suppresses the inflammation, its action on arthritis is investigated in vivo through PG-PS model of arthritis. PG-PS is a very crucial structural component of Gram-positive bacterial cell walls and it causes rheumatoid like arthritis in rats. In rats injected with PG-PS which suffer from infiltration of inflammatory cells, synovial hyperplasia, edema, and ankle swelling, these effects of PG-PS model of arthritis can be reduced by glycine supplementation [66].

### 5.7. Cancer Therapy: Glycine

Polyunsaturated fatty acids and peroxisomal proliferators are very good tumor promoters as they increase cell proliferation. Kupffer cells are very good sources of mitogenic cytokines such as TNF*α*. Glycine taken in diet can suppress cell proliferation caused by WY-14,643 which is a peroxisomal proliferator and by corn oil [67, 68]. The synthesis of TNF*α* by Kupffer cells and activation of nuclear factor *κ*B are blocked by glycine. The 65% of tumor growth of implanted B16 melanoma cells is inhibited by glycine indicating that glycine has anticancer property [69].

### 5.8. Role of Glycine in Vascular Health

One of the researchers demonstrated that platelets express glycine gated chloride channels in rats. They also reported that human platelets are glycine responsive and express glycine gated chloride channels [70]. Zhong et al. (2012) have reported that preadministration of 500 mg/kg of glycine could reduce cardiac ischemia reperfusion injury [71]. One of the researchers demonstrated that 3 mM of glycine supported enhanced survival rate of in vitro cardiomyocytes and later subjected to one hour of ischemia and thereafter reoxygenated. 3 mM of glycine was also protective for cardiac ischemia reperfusion ex vivo model [72]. Sekhar et al. reported that glycine has an antihypertensive effect in sucrose fed rats [73, 74].

## 6. Conclusion

Glycine has a wide spectrum of defending characteristics against different injuries and diseases. Similar to many other nutritionally nonessential amino acids, glycine plays a very crucial role in controlling epigenetics. Glycine has much important physiological function in humans and animals. Glycine is precursor for a variety of important metabolites such as glutathione, porphyrins, purines, haem, and creatine. Glycine acts as neurotransmitter in central nervous system and it has many roles such as antioxidant, anti-inflammatory, cryoprotective, and immunomodulatory in peripheral and nervous tissues. Oral supplementation of glycine with proper dose is very successful in decreasing several metabolic disorders in individuals with cardiovascular disease, various inflammatory diseases, cancers, diabetes, and obesity. More research investigations are needed to explore the role of glycine in diseases where proinflammatory cytokines, reperfusion or ischemia, and free radicals are involved. Mechanisms of glycine protection are to be completely explained and necessary precautions should be taken for safe intake and dose. Glycine holds an enormous potential in enhancing health, growth, and well-being of both humans and animals.
