# Corrigendum to “Multifarious Beneficial Effect of Nonessential Amino Acid, Glycine: A Review”

**DOI:** 10.1155/2022/9857645

**Published:** 2022-02-23

**Authors:** Meerza Abdul Razak, Pathan Shajahan Begum, Buddolla Viswanath, Senthilkumar Rajagopal

**Affiliations:** ^1^Department of Biochemistry, Rayalaseema University, Kurnool 518002, India; ^2^Department of Zoology, K.V.R. Govt College for Women, Kurnool 518002, India; ^3^Department of Bionanotechnology, Gachon University, San 65, Bokjeong Dong, Sujeong Gu, Seongnam Si, Gyeonggi Do 461 701, Republic of Korea

In the article titled “Multifarious Beneficial Effect of Nonessential Amino Acid, Glycine: A Review” [1], there was a typographical error in Section 3.2, where the word “heroin” should be corrected to “threonine.”

## References

[1] Razak M. A., Begum P. S., Viswanath B., Rajagopal S. (2017). Multifarious Beneficial Effect of Nonessential Amino Acid, Glycine: A Review. *Oxidative Medicine and Cellular Longevity*.

